# Towards Better Oral Health Prevention: the Value for Money of Fluoride Toothpaste

**DOI:** 10.1093/eurpub/ckac131.026

**Published:** 2022-10-25

**Authors:** T Hofbauer, J Coughlan, J Gierth, N Gkekas, S Listl, M Pattamatta, A Põld

**Affiliations:** 1 University of Heidelberg, Heidelberg, Germany; 2 NHS Foundation, London, UK; 3 University of York, York, UK; 4 University of Radboud, Nijmegen, Netherlands

## Abstract

**Background:**

Although largely preventable, tooth decay (dental caries) is among the most common health conditions worldwide and contributes to substantial dental treatment expenditures. While Fluoride Toothpaste (FT) is considered an essential strategy for oral health prevention, its market price has been shown to vary substantially across various settings. Against this background, the present study aimed to develop a decision analytical model to evaluate the cost-effectiveness of FT in various settings.

**Methods:**

Leaning on WHO CHOICE methodology, evidence scoping and an expert consensus were facilitated to extract model input parameters which were then fed into cost-effectiveness-analyses (CEA) for FT. The cost-effectiveness of the interventions was expressed as cost per Disability-Adjusted Life Year (DALY) averted.

**Results:**

The CEA identified a high likelihood for FT to be a cost-efficient treatment strategy in settings with comparably low market prices for FT. FT was less likely to be a cost-efficient treatment strategy in settings with comparably high market prices for FT.

**Conclusions:**

The developed decision analytical model is suitable to inform policymakers about the extent to which FT represents good value-for-money under different market prices.

**Key messages:**

• Fluoride toothpaste can provide high value for money as an oral health preventive strategy.

• Policymakers need to ensure the affordability of fluoride toothpaste in order to harvest relevant efficiency gains.

